# Recovery of Dissolved Hydrogen Sulfide from Various Wastewater Streams Using Membranes and Other Relevant Techniques: A Review

**DOI:** 10.3390/membranes13070646

**Published:** 2023-07-05

**Authors:** Abdul Waheed, Isam H. Aljundi, Umair Baig

**Affiliations:** 1 Interdisciplinary Research Center for Membranes and Water Security, King Fahd University of Petroleum and Minerals, Dhahran 31261, Saudi Arabia; aljundi@kfupm.edu.sa; 2 Chemical Engineering Department, King Fahd University of Petroleum and Minerals, Dhahran 31261, Saudi Arabia

**Keywords:** dissolved H_2_S, hydrophobic membrane, H_2_S scavengers, membrane contactors, H_2_S diffusion, wastewater treatment

## Abstract

Given the significance of dissolved H_2_S, various techniques have been explored in the literature. The current review describes in detail the various membrane-based techniques, such as membrane contactors, for removing dissolved H_2_S from various wastewater streams. Various types of hydrophobic membranes have been used, with more emphasis placed on PVDF hollow fiber membranes. The hydrophobic membranes do not allow water to pass through, whereas H_2_S is readily allowed to pass through the membrane at ambient conditions. In addition, the use of monoethanol amine triazine (MEA-Triazine)- based H_2_S scavengers has also been described in detail, including the possible scavenging mechanism. The possibility of different types of byproducts has also been explained along with the possible routes to get rid of scavenger byproducts, such as apDTZ. The use of peroxy acetic acid has also been explained to oxidize and solubilize apDTZ. Furthermore, the use of vacuum-based dissolved H_2_S gas has also been described in detail. The application of the Knudsen and bulk diffusion models to the separation of dissolved H_2_S through the pores of the hollow fibers has also been explained. Finally, the future challenges and possible solutions along with concluding remarks have also been mentioned in the current review.

## 1. Introduction

Wastewater is generated from several sources and industries. Sewage wastewater is released in immense quantities from domestic facilities [1]. In one instance, the increase in sewage wastewater reflects an increase in human population over time. Similarly, industrial zones also release huge quantities of wastewater. The oil and gas industry also releases enormous quantities of sour wastewater [2]. Produced water (PW) is another stream generated during the drilling for oil in the oil fields [3,4,5]. The fishing industry and sugarcane processing industries also produce large quantities of wastewater [6]. These wastewater streams contain several pollutants, of which dissolved H_2_S is one of the most serious [7,8].

Dissolved H_2_S is also found in several natural water bodies, such as thermal springs and groundwater aquifers [9,10,11]. Similarly, the presence of thiosulfate in the agricultural wastewater also results in the production of some quantities of dissolved H_2_S in the revisers and lakes [12,13]. In addition, many industries, including paper and pulp, tannery products, catalytic cracking of crude oil, and the release of huge quantities of effluents laden with H_2_S are also sources of dissolved H_2_S in water bodies [14,15]. H_2_S has also been known to be a cause of corrosion in pipelines that are used for water transmission. The oxidation of H_2_S leads to the production of sulfuric acid in the pipelines, which promotes corrosion. Where H_2_S is highly toxic to human beings, it also has a detrimental effect on aquatic life. Hence, different countries have set different limits for the release of dissolved H_2_S in their water bodies. In this regard, the World Health Organization (WHO) has set 0.05 ppm as a permissible limit for drinking water and declared 10 ppm as toxic [16]. 

Another cause of concern is the presence of H_2_S in the feed of RO plants, leading to membrane fouling. In a study conducted by Kinser et al. [17], it was found that H_2_S is commonly found in the Florida aquifers and Lower Hawthorn aquifers. It has been determined that the major fouling of the RO membranes is caused due to elemental sulfur, sulfur oxides, and metal sulfides generated during the oxidation of H_2_S, as shown in the following equation as per American Water Works Association (1990):

2H_2_S + O_2_ ⟶ 2H_2_O + S_(s)_ (1)

Hence, the presence of dissolved H_2_S in the feed water is a cause for concern for the life and performance of the RO membrane. Basically, the chemistry of H_2_S is highly dependent on the pH of the aqueous medium. Snoeyink and Jenkins equations for the dissociation of H_2_S in water are given below in Equations (2) and (3):

H_2_S ⇔ H^+^ + HS^−1^………………… (pK_1_ = 7.0) (2)

HS^−^ ⇔ H^+^ + S^−2^……………….... (pK_1_ = 12.0) (3)

It is clear from the above-mentioned equations that H_2_S dissociates in two ways. In the first step, the H_2_S gas is dissociated to generate bisulfide ions (HS^−^^1^), which are further dissociated into sulfide ions (S^2−^).

**Figure 1:**
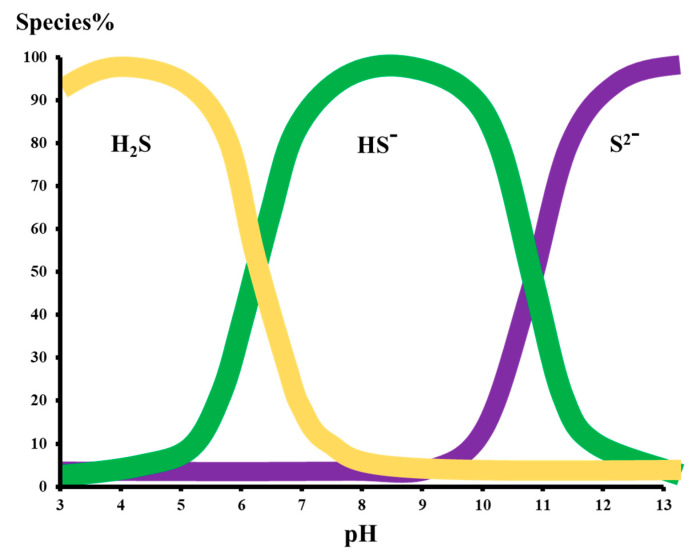
The H_2_S species present in an aqueous medium at changing pH [17].

As the pH of the aqueous medium decreases below 7, the sulfide ion is present in the form of H_2_S gas. However, as the pH increases, the H_2_S is present in dissociated forms either as HS^−1^ or S^−^^1^. As the pH reaches the ambient pH of 7, 50% H_2_S is present in the gas form while H_2_S is almost completely converted into a bisulfide ion as pH reaches 9 to 10. However, as the pH reaches 11.8 to 12.0, the bisulfide and sulfide ions exist in equilibrium while at pH 13–14, the majority of the H_2_S is converted into sulfide ions. These findings have been presented in the following Figure 1.

It has been found that, at pH 5.1, H_2_S is 100 percent present in gas form. Hence, the pH of the feed at an RO plant is highly important as it determines the fate of the gases during permeation through the membranes. At a lower pH, gases such as H_2_S and CO_2_ can readily permeate through the RO membranes and, hence, require post-treatment for degasification of the product clean water. However, at higher pH values, the dissolved sulfide ions are rejected up to 93% by the RO membranes. Hence, depending on the pH of the stream, sulfide ions can be present either in the concentrate or permeate. Hence, based on the content of the dissolved oxygen, the sulfide ions are oxidized to elemental sulfur leading to membrane fouling as depicted in Equation (1), which is difficult to remove.

The undesirable components such as H_2_S and CO_2_ present in natural gas upon dissolution in water become exceedingly corrosive to the gas transmission system and compromise its integrity. Generally, conditioning is applied to mainly remove H_2_S, CO_2_, water and solids. However, H_2_S being highly flammable, toxic, and heavier than air can accumulate at dangerous levels in pipes, valves, and tanks [18]. The mere presence of 0.5 mg L^−^^1^ of H_2_S in the potable water is noticeable while 1.0 mg L^−^^1^ of dissolved H_2_S feels quite offensive. The rotten egg smell is also attributed to H_2_S generated due to bacterial action on the sulfates present in the wastewater streams. The H_2_S taste can be detected at a very low concentration of 0.05 mg L^−^^1^ [19]. H_2_S is produced in wastewater through the microbial action of two commonly known bacterial strains, such as Desulfovbrio desulfuricans and Desulfotomaculum, which carry out the reduction of sulfates under anaerobic conditions [20]. Similarly, other bacterial strains, such as Dimethylpolysulfides and Methylmercaptan, are also involved in production of sulfur-containing compounds. Many types of Pseudomonades are also responsible for producing sulfur-containing compounds [21]. Hence, the presence of dissolved H_2_S is highly deleterious to not only living beings, but also to wastewater treatment and transmission systems.

Given the huge significance of dissolved H_2_S as a cause of concern for not only the wastewater treatment facility, but also the community living nearby, there is a desperate need to separate and wisely use it for useful purposes. Several reports have been published in the literature regarding the separation of dissolved H_2_S from wastewater streams. Researchers have explored the potential of membranes, H_2_S scavengers, and other relevant technologies to remove and recover the dissolved H_2_S from wastewater streams. Most of the reviews published in the literature have primarily focused on the separation of H_2_S gas from a gaseous mixture. Hence, the current review has covered the literature related to the removal of dissolved H_2_S from wastewater.

## 2. Approaches to Treat H_2_S

**Figure 2:**
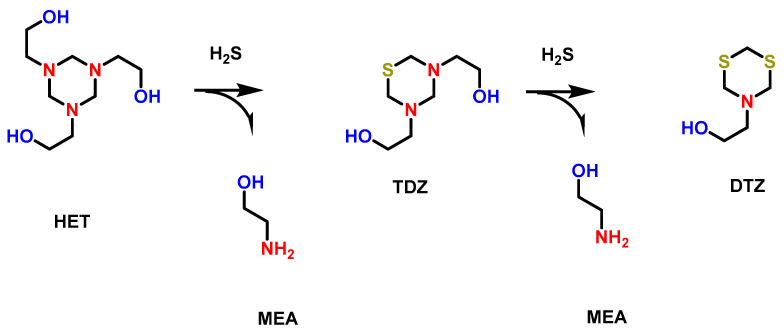
Scavenging mechanism of H_2_S by HET scavenger and production of MEA and DTZ.

Among the several industries affected by H_2_S, the oil and gas industry is highly impacted by the presence of H_2_S gas due to its toxicity and, above all, the corrosion of the pipelines during the transmission of the gases [22]. In order to get rid of H_2_S (gas sweetening), certain chemical scavengers, such as triazine-based compounds, are added to the feed to convert the H_2_S into organic molecules containing S 1,3,5-tri(2-hydroxyethyl)hexahydro-S-traizine (HET), which is added to the H_2_S gas stream as a basic solution to absorb H_2_S and convert it into non-harmful products. As demonstrated in the following Figure 2, two nitrogen atoms of HET are replaced by S atoms of H_2_S, yielding the chemically essential byproduct monoethanolamine and spent HET called 5-(2-hydroxyethyl)hexahydro-1,3,5-dithiazine (DTZ) [23,24].

HET is generally added in excess equivalence in comparison to H_2_S, which ensures the complete degradation of H_2_S so that H_2_S concentration reaches permissible discharge limits as per the regulations of government agencies. Although HET is an effective H_2_S scavenger that has been efficiently used during the treatment of wastewater of offshore oil and gas facilities, HET and its byproducts (MEA and DTZ) are considered environmental hazards that must be removed by the discharge of the treated effluent into the environment.

To understand the chemistry and fate of the byproducts, a detailed study on the mechanism of the triazine scavenging reaction was carried out. This study also proposed chemical means for the removal of scavenger byproducts. In fact, the scavenger initial byproducts lead to the formation of heavy and insoluble deposits that become difficult to remove. The scavenger byproduct deposits are shown below in Figure S1 [25].

Therefore, a thorough understanding of the chemistry and mechanism of the formation of these byproducts was studied. Generally, it is believed that the byproduct is 1,3,5-triatiane, in which all the nitrogen atoms of triazine ring are replaced by S atoms [18,26,27]. However, it has been established in the literature that the actual byproduct generated is dithiazine not 1,3,5-triatiane. Hence, the deposits seen are basically dithiazine. Figure S2 shows the mechanism of reaction of triazine scavengers. Protonation of the nitrogen atom generates the site for the nucleophilic substitution reaction of bisulfide (HS^-^). The electronegativity of the two nitrogen atoms linked with carbon atoms generate an electropositive carbon atom in the triazine molecule. The existence of an electron deficient carbon atom is an ideal site for a nucleophilic substitution reaction with HS^-^. Upon the nucleophilic substitution (SN_2_) reaction with HS^-^, an amine molecule is released as a leaving group. The resultant compound of the first SN_2_ reaction produces thiadiazene (Figure S2a). The second SN_2_ reaction produces the dithiazine that has two sulfur atoms and one nitrogen atom (Figure S2b). The GC-MS analysis of the reaction mixture showed the presence of dithiazine. However, no evidence was collected showing the existence of 1,3,5-trithiazine. The possibility of a nucleophilic attack of HS^-^ on the dithiazine ring is not possible and, hence, the 1,3,5-trithiazine ring is not formed as a byproduct. This possibility of a reduced attack arises since a carbon atom is not connected to two adjacent nitrogen atoms in dithiazine. Instead, a carbon atom is linked with one nitrogen and one sulfur atom, and hence, the carbon atom is not as electron-deficient as in thiadiazine. Hence, the attack of HS^-^ on dithiazine is not possible, as shown in Figure S2c. Molecular electrostatic potential (MEP) measurements showed the presence of a more positive charge on carbon in the presence of a nitrogen atom. Upon the inclusion of a sulfur atom, the positive charge is reduced, and hence, the nucleophilic substitution reaction is not possible (Figure S2d) [28].

Similarly, further investigations have shown that the use of ethanol amine has potentially resulted in the generation of amorphous polymeric dithiazine (apDTZ) starting from dithiazine. Various methods have been developed, including the use of cosolvents and hydrogen peroxide. However, these methods have not proved sufficient to remove the apDTZ. However, the use of organic per(oxy) acids has proven highly efficient for removing the apDTZ. The use of per(oxy) acetic acid has also emerged, leading to the digestion of insoluble polymeric apDTZ. It has been found that the S in apDTZ is destined for SO_4_^2−^, whereas the carbon is converted into formic acid and formats.

**Figure 3:**
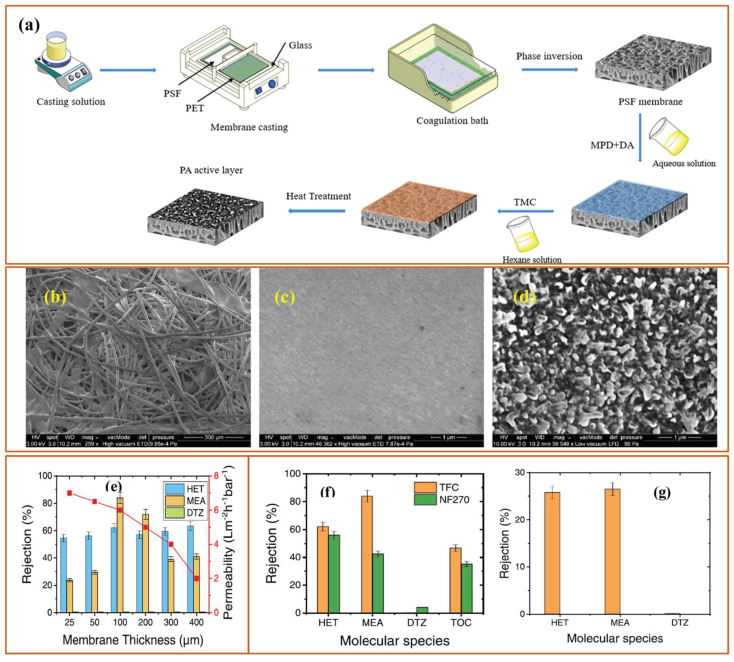
(**a**) A schematic representation showing fabrication of TFC membranes, SEM micrographs of (**b**) PET, (**c**) PSF, and (**d**) TFC membranes and rejection of main compounds of the SUS solution using the NF270 and the synthesized TFC membrane; (**e**) Effect of membrane thickness on the rejection of HET, MEA and DTZ and permeability of the synthesized membrane (measured at permeate recovery of 50%), (**f**) Rejection of main compounds of the SUS solution using the NF270 and the synthesized TFC membrane and (**g**) Rejection of HET, MEA and DTZ by the synthesized TFC membrane in single-solute solutions prepared with 1 g/L of HET, 10 g/L of MEA and 50 mg/L of DTZ in Milli-Q water at 40 °C, 100 µm membrane thickness, and 50% recovery [29].

Hence, M.N. Fini et al. used a hybrid membrane system consisting of nanofiltration (NF) and reverse osmosis (RO) membranes to reduce the total organic content (TOC) of spent and unspent scavenger (SUS) wastewater. The NF270 membrane was able to reduce TOC up to 65% with separation of HET from DTZ with 70% removal of HET and zero removal of DTZ. Then, the permeation of NF270 was used as a feed for the XLE RO membrane where the effluent stream was discharged with a 98% removal of TOC [24]. The same group also fabricated a thin film composite (TFC) polyamide membrane through interfacial polymerization (IP) by using a mixture of meta-phenylenediamine (MPD) and dopamine hydrochloride (DA) as an aqueous phase while trimesoyl chloride (TMC) as the non-aqueous phase. The membrane MPD-DA/TMC polyamide active layer was deposited on the polysulfone (PSf)/polyethylene terephthalate (PET) support. The following Figure 3a shows the schematic representation of IP for membrane fabrication along with structural (Figure 3b–d) features and filtration performance (Figure 3e–g).

Figure 3a shows the wet phase inversion followed by IP between MPD-DA and TMC solutions while Figure 3b shows a SEM micrograph of PET where the fibers of PET can be clearly seen. After the phase inversion, the PSf layer covered the PET support (Figure 3c), which has a highly microporous structure. However, after IP, the polyamide active layer showed a completely altered surface morphology with a highly dense layer, leading to a polyamide TFC membrane. Afterward, the performance of the membranes was tested to remove the by-products of dissolved H_2_S scavengers, such as HET, MEA, and DTZ. Figure 3e shows the effect of membrane thickness on the rejection and permeability of the membrane. As the membrane thickness increases, the permeability decreases, which can be attributed to the increased mass transfer resistance during the permeation of water through the membranes. However, the rejection increases with increasing membrane thickness, reaching maximum rejection at 100 µm and 200 µm. The MPD-DA/TMC membrane showed a rejection of 62% HET and 82% MEA with no rejection of DTZ while the commercial nanofiltration membrane NF270 showed a rejection of HET 56% and MEA 43% and 4%, respectively. Figure 3g shows the rejection performance of 100 µm membrane when separate solutions of each pollutant were used during filtration experiments [29].

Although HET has shown a huge potential for removing dissolved H_2_S from wastewater, the removal of by-products, such as DTZ and MEA containing removed H_2_S, needs a highly dense membrane. Similarly, other methods to treat dissolved H_2_S, such as the use of oxidizing agents, such as oxygen, hydrogen peroxide, hypochlorite, and chlorine, result in the production of hazardous by-products. Hence, alternative ways of treating dissolved H_2_S are being explored by the research community. In one such effort, G.K. Agrahari et al. used hollow fiber membrane contactors (HFMCs) to remove dissolved H_2_S from wastewater streams. The removal of dissolved H_2_S was based on the principle of interphase mass transfer. Interphase mass transfer offers high contact between the two phases without mixing, which maximizes the transfer of the dissolved H_2_S from the wastewater streams to the permeate side. The pressure drops between the two independently flowing phases are also minimal. Furthermore, the ease of operation, highly compact design, ease of flow controls along with low cost, and small footprint make the HFMC technique a potential technology for treating dissolved H_2_S.

**Figure 4:**
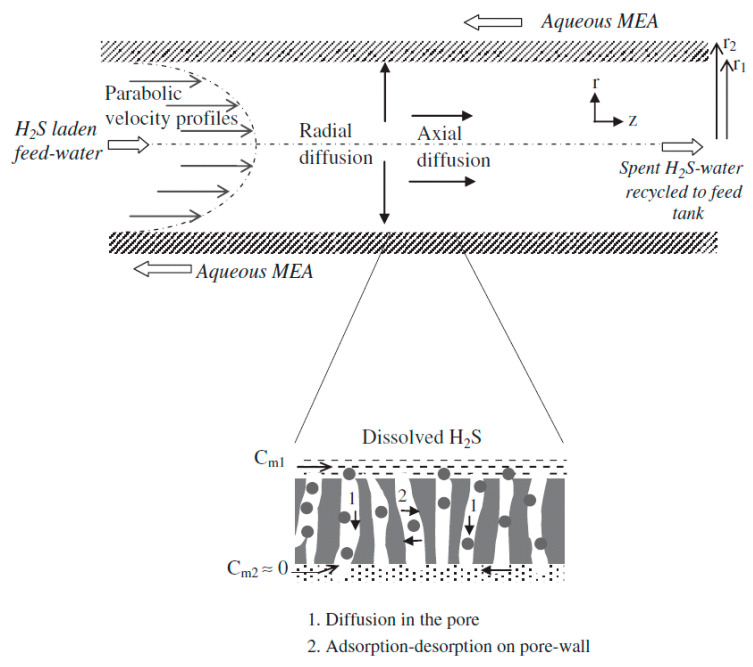
Schematic of transport steps governing H_2_S removal from water using the HFMC module. Adapted with permission from Ref. [31] (2013, Elsevier).

Figure 4 shows an illustration explaining the steps responsible for the removal of dissolved H_2_S from the feed using HFMC. The hollow fiber membrane was fabricated using polypropylene (PP) as a hydrophobic membrane [30] with microspores in the matrix of the membrane. During the H_2_S removal experiment, the H_2_S-laden feed water was passed through the lumen of the hollow fiber while the monethanolamine (MEA) solution was used as an extractant on the shell side, flowing in a countercurrent manner to the feed. The dissolved H_2_S is desorbed from the feed and enters the pores of the PP membrane. Hence, the dissolved H_2_S is separated from the aqueous phase and enters the polymeric phase. The wetting of the membrane with water is negligible as PP is a hydrophobic polymer. The dissolved H_2_S is desorbed from the feed and adsorbed onto PP, and hence, the transfer of H_2_S from feed to extractant is dependent upon the adsorption–desorption of H_2_S inside the micro pores of PP. On the extractant side, a rapid reaction occurs between MEA and H_2_S, and hence, the concentration of H_2_S on the feed’s side decreases and reaches equilibrium with the extractant [31].

**Figure 5:**
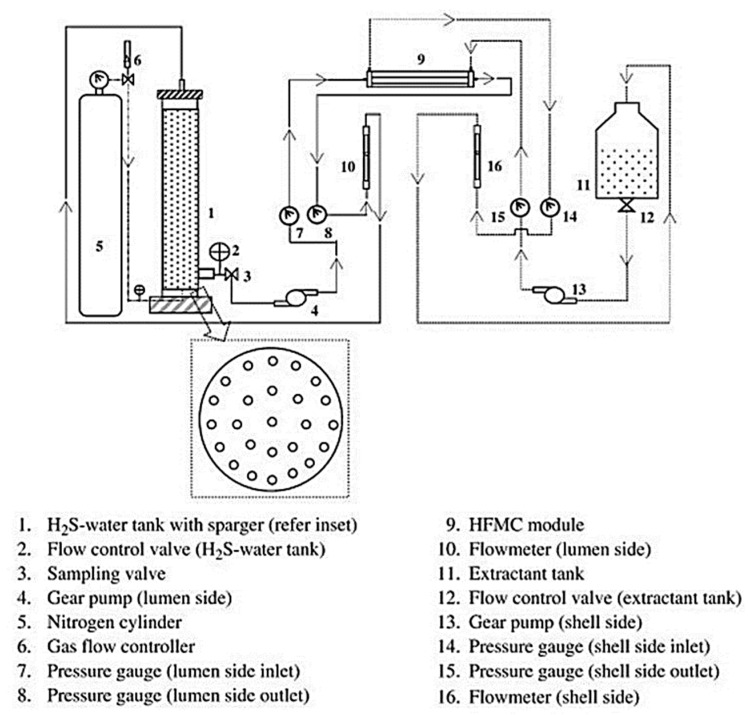
Scheme of experimental setup fabricated and used for H_2_S removal from water using the HFMC module. Sparger was fitted at the base of feed tank for purging water with N_2_ gas. Adapted with permission from Ref. [31] (2013, Elsevier).

Hence, the use of HFMC with a hydrophobic membrane proved to be highly useful for removing dissolved H_2_S from the wastewater. The concentration of dissolved H_2_S in the feed ranged from 300 ppm to 1600 ppm. The HFMC reached a removal efficiency of 98%. The following Figure 5 shows the flow diagram of the HFMC membrane testing system.

**Figure 6:**
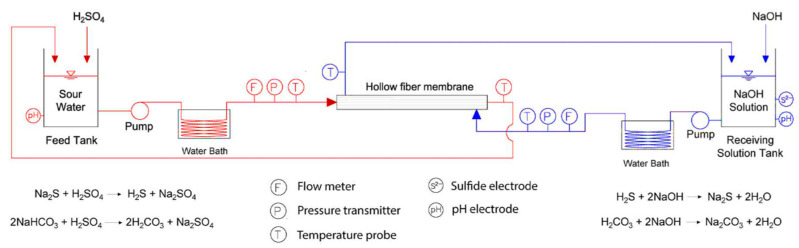
Bench scale system schematic. Adapted with permission from Ref. [32] (2017, Elsevier).

In a similar work, Minier-Matar et al. also used membrane contactors for removing dissolved H_2_S from the sour water stream obtained from a natural gas onshore processing facility with an S^2−^ ion concentration of 100 mg L^−^^1^. A hollow fiber polypropylene (PP) crossflow membrane module was used in this study. The membrane module was installed in the custom-made filtration system shown below in Figure 6. Figure 6 shows that the hollow fiber membranes were supplied with a sour water feed containing dissolved H_2_S, where sulfuric acid was used to keep an acidic pH in the feed tank while NaHCO_3_ was used to study the impact of CO_2_ on the removal of dissolved H_2_S from the feed tank. A NaOH-receiving solution was flown in a counter-current manner for removing dissolved H_2_S and the subsequent reaction yielding Na_2_S, which is a harmless species, and its concentration was measured to know the S^2−^ ion concentration in the receiving solution.

**Figure 7:**
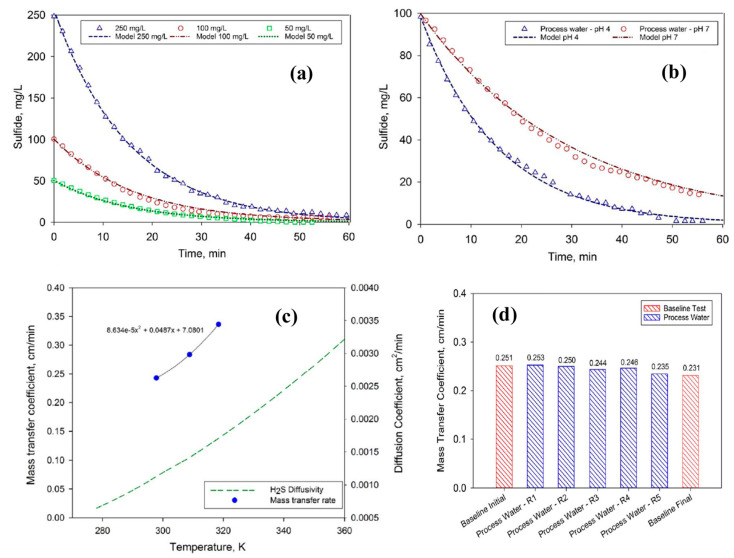
(**a**) Sulfide concentration in the feed solution as function of time for three different initial H_2_S concentration—Synthetic solution, (**b**) Sulfide concentration in the feed solution as function of time at pH 4 and 7—Gas field process water, (**c**) mass transfer coefficient as function of temperature (experimental data) compared to H_2_S diffusivity in water (based on OLI electrolyte simulations), and (**d**) change in the mass transfer coefficient during fouling experiments [32]. Adapted with permission from Ref. [32] (2017, Elsevier).

As the membrane in the current study was hydrophobic in nature and the temperature on both the feed side and the receiving solution side was the same, which ensured that unlike membrane distillation (MD), no water vapors were permeated through the PP membrane. Hence, membrane contactors do not allow water but only H_2_S to transfer through the pores of the PP membrane. Minier-Matar et al. also studied the kinetics of mass transfer of H_2_S through a PP membrane and found that the transfer of the H_2_S from a sour-water boundary was the rate limiting step. In addition, different parameters, such as effect of S^2−^ ion concentration, feed pH, temperature, and fouling studies, were also carried out as given in the following Figure 7. The mass transfer co-efficient “K” was determined for different initial concentrations of dissolved H_2_S, which were 50 mg L^−^^1^, 100 mg L^−^^1^, and 250 mg L^−^^1^ at a pH of 4 to ensure that all dissolved S^2−^ ions was in the form of H_2_S. The calculations found values of K as 0.240, 0.246, and 0.243 cm min^−^^1^ for 50 mg L^−1^, 100 mg L^−1^, and 250 mg L^−1^, respectively. The findings revealed that the mass transfer co-efficient was independent of initial concentrations of dissolved H_2_S (Figure 7a). In the case of pH effect, the removal of H_2_S was found to 100% cat pH 4 compared to pH of 7 (Figure 7b). In case of increasing temperature of the feed, the mas transfer was found to be exponentially increasing (Figure 7c). The fouling performance of the PP membrane was also studied by using a feed of 2 g L^−1^ of dissolved H_2_S where it was found that the mass transfer co-efficient was decreased by 8% owing to the fouling of the membranes (Figure 7d) [32].

**Figure 8:**
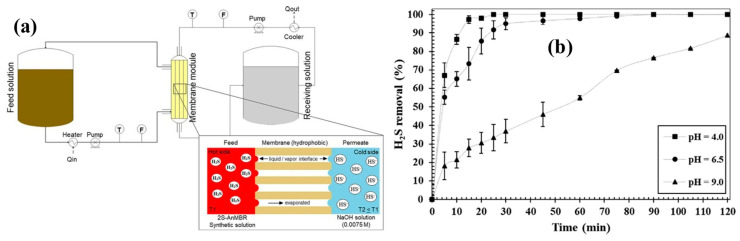
(**a**) Schematic diagram of system used and (**b**) H_2_S removal at pH 4.0, 6.5, and 9.0 (Tfeed = 40 °C; Treceiving = 25 °C; Q = 0.3 L min^−1^, synthetic solution [H_2_S] =166 ± 15 mg L^−1^) [33]. Adapted with permission from Ref. [33] (2020, Elsevier).

In a similar application, Silva et al. used membrane distillation (MD) for removing H_2_S from the permeate of an anaerobic bioreactor. Although an anaerobic membrane bioreactor has proven efficient in producing high-quality effluent with a tolerance for variation in feed quality; one of the disadvantages is treating the wastewater rich in sulfates and organic matter. Since the concentration of sulfates and organic matter is higher in the feed, the sulfate-reducing bacteria can use sulfate as a substrate resulting in the production of H_2_S. Hence, the removal of dissolved H_2_S is required to reuse the water. Hence, the Silva group designed a modified direct contact membrane distillation (M-DCMD) configuration for removal of dissolved H_2_S from the real wastewater (Figure 8). The real wastewater was the permeate of the two-stage anaerobic membrane bioreactor (2S-AnMBR). The dissolved H_2_S (166 ± 15 mg/L^−1^)-containing permeate was generated due to the anaerobic biological digestion of sugarcane vinasse collected from the local company. The following M-DCMD system having a polypropylene (PP) hollow fiber membrane was used for removing H_2_S. In this M-DCMD system, the dissolved H_2_S feed was passed through the lumen of the hollow fiber membrane while a cold NaOH-receiving solution was passed on the shell side of the membrane. As PP is a super-hydrophobic material in nature, the entry of water into the membrane pores is not possible. H_2_S speciation at different pH values affects the solubility of H_2_S in the feed, which affects the content of S^−2^ ions in the feed, and hence, the removal of dissolved H_2_S from water is dependent upon the pH of the feed solution. At pH 4, 100% of H_2_S is present in a gaseous state, which increases the removal rate of H_2_S (Figure 8b). However, as the pH increases from 6 to 9, the H_2_S gets dissolved in water and is found in dissolved S^−2^ form. Hence, the flux of H_2_S was found to be 0.00638, 0.00340, and 0.00109 Kg H_2_S m^−2^ h^−1^ at pH 4, 6.5, and 9, respectively.

An elaboration of the structure of the PP hollow fiber membrane is given in the inset in Figure 8a, which shows how the hydrophobic PP membrane acts as a separation between the H_2_S-containing feed and the NaOH-receiving solution. The PP membrane being hydrophobic does not allow the water to pass through the membrane from either solution while H_2_S diffuses from the feed to the receiving solution. Moreover, the two solutions move in a counter-current manner, which increases the rate of removal of H_2_S from the wastewater stream [33]. M-DCMD uses a hot feed at 50 °C compared to membrane contactors, which do not involve a hot feed and instead operate at room temperature.

**Figure 9:**
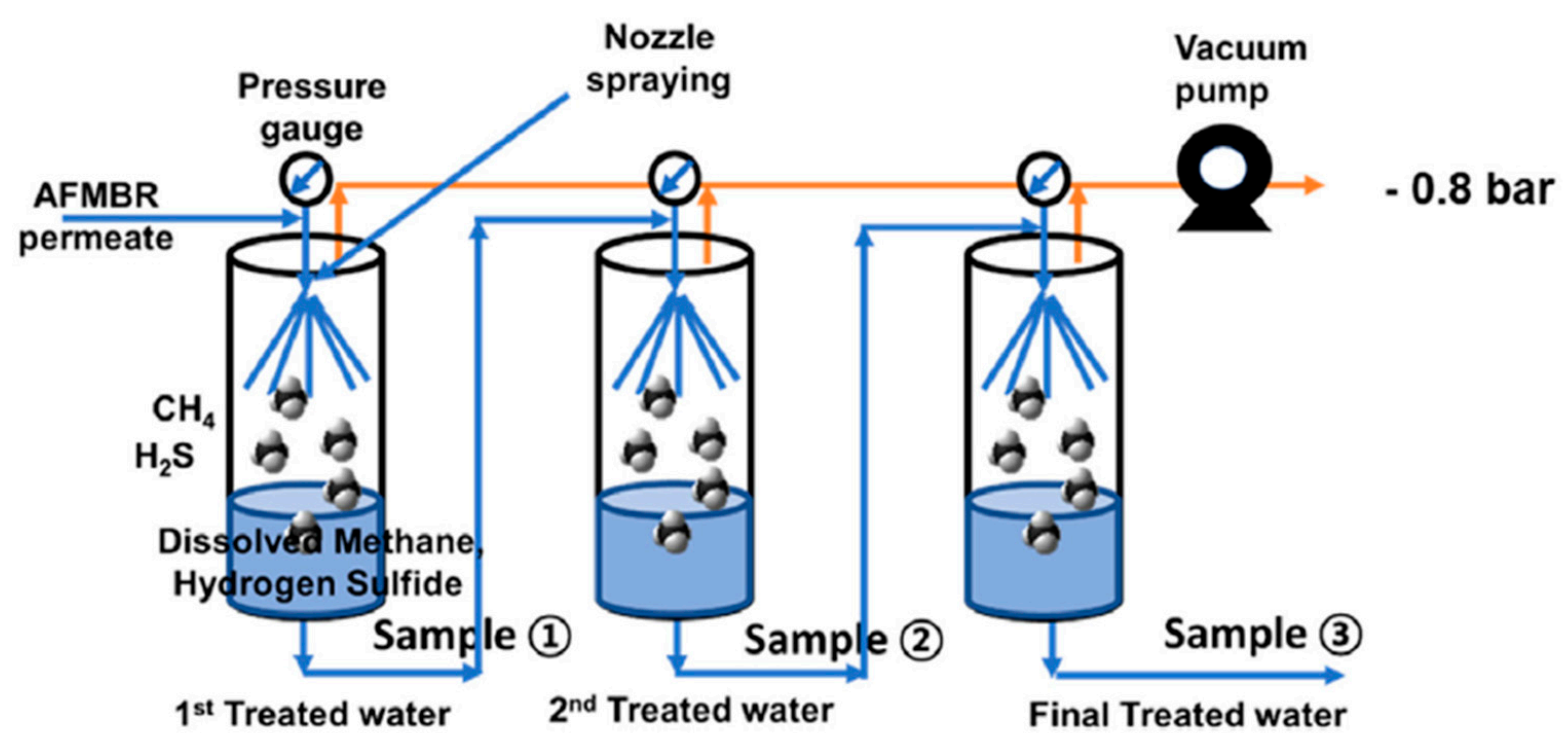
Schematic diagram of the three-stage vacuum degasifier system (nozzle type) [35]. Adapted with permission from Ref. [35] (2020, Elsevier).

Like other wastewater streams, the permeate of the anaerobic membrane bioreactor has also been identified to possess considerable quantities of dissolved H_2_S in addition to dissolved CH_4_. In the case of the anaerobic digestion of sludge, sulfate-reducing bacteria take over methanogenic bacteria, leading to the production of H_2_S. Since H_2_S has high water solubility, it is found in a dissolved form in the permeate, causing a foul smell, corrosion, SOx production, and toxicity. It has been reported that a concentration of 60 mg L^−^^1^ of H_2_S can cause an irreversible process failure of both acetate-fed and propionate-fed chemostats in an anaerobic digester [34]. Although hydrophobic degassing membranes have been found to be highly useful for removing dissolved gases, some issues, such as mass transfer resistance and membrane fouling, need to be optimized. Hence, there is a need for discovering innovative and more promising routes and techniques for removing dissolved H_2_S. In one such effort, E. Lee et al. developed a staged vacuum-based degasifier system for removing dissolved gases, including H_2_S from the permeate of a staged anaerobic fluidized membrane bioreactor (SAF-MBR) as given in the following Figure 9. Three stainless steel chambers are connected in a series with the vacuum pump, and the influent is entered in chamber 1 at stage 1 in the form of a spray through a nozzle at 15 mL s^−^^1^ sequentially. A vacuum of −0.8 bar was applied across the chamber by the vacuum pump.

**Figure 10:**
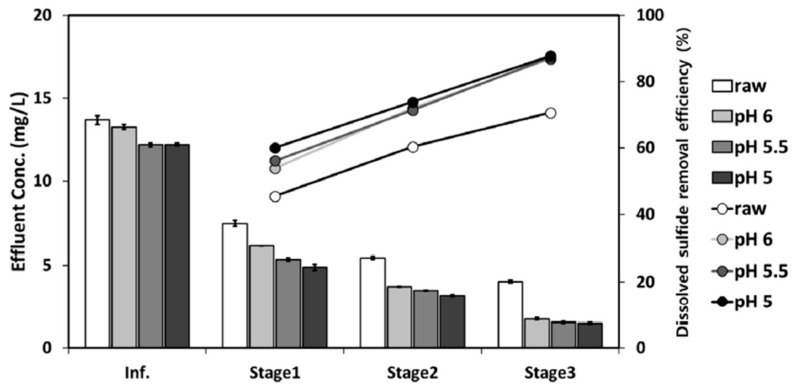
Performance of a three-stage vacuum degasifier for dissolved H_2_S removal. Adapted with permission from Ref. [35] (2020, Elsevier).

The degasifier system resulted in a considerable decrease in the concentration of pollutants as the COD was recorded to decrease as the influent was moved from stage 1 to stage 3. In the case of the pH-regulated stream, the removal was found to be higher than the raw stream. Similarly, the removal efficiency of H_2_S was found to increase while moving from stage 1 to 2 and finally to stage 3. As the pH decreases and moves towards an acidic region, the H_2_S removal efficiency increases as shown in Figure 10. The removal of H_2_S reached 88% with pH-adjusted samples. This study found that dissolved H_2_S removal was governed by factors, such as pH, contact time, temperature, turbulence of the liquid phase, and equilibrium constant [35].

## 3. Mathematical Analysis for the Removal of Dissolved H_2_S

For the sake of better understanding and improving the performance of membrane-based separations of dissolved H_2_S, mathematical modeling has been carried out in the literature. The researchers have used a hollow fiber membrane contactor with liquid–liquid extraction mode using a hydrophobic membrane as has been discussed in the previous sections. The wastewater stream was introduced into the lumen side of the membrane while an extractant solution was passed in a counter-current manner on the shell side of the hollow fiber membrane. The gas is desorbed from the lumen side and passed to the shell side of the membrane where it is instantaneously reacted with the extractant which is either NaOH or ethanolamine solution. The mathematical models were developed based on the radial and axial diffusion of H_2_S. The mass transfer across the pores of the membrane was explained based on Knudsen and bulk flow. The following assumptions have been made for the mathematical models:

1. Given the dilute nature of the feed solution, the physical and transport conditions for the feed solution remain unchanged during gas the separation experiment;
2. The isothermal operating conditions have no change in liquid density;
3. There is no wetting or swelling of the membrane, and pores are assumed to be filled with air;
4. There is a fully developed laminar flow with parabolic velocity profiles on the lumen side of the membrane. The operational experimental conditions for flow on the lumen side are chosen to be at a very small Reynold number, NRe, which is generally calculated to be 3;
5. The pH of the feed solution is controlled so that H_2_S remains in molecular form;
6. Two-dimensional unsteady state flow with axial and radial diffusion of H_2_S molecules in the lumen of the membrane;
7. The volumes of feed and extractant solutions used during H_2_S removal are large compared to the volume of membrane contactors;
8. An instantaneous reaction takes place on the shell side as the extractant phase is highly concentrated.

### Equations for the Lumen Side of the Membrane

The gas transport on the lumen side is expressed by an unsteady state convective-diffusion equation, which accounts for both radial and axial diffusion components as given below:

(4) $$\frac{\partial C_{l}}{\partial t}+\nu_{z}\frac{\partial C_{l}}{\partial_{z}}=D_{l}\left(\frac{1}{r}\;\frac{\partial }{\partial r}\;\left(\frac{\partial C_{l}}{\partial r}\right)+\frac{\partial^{2}C_{l}}{\partial z^{2}}\right)\ldots$$

where *C_l_* and *D_l_* are the concentration (moles m^−3^) and diffusivity (m^2^ s^−1^) of H_2_S in the feed. The laminar flow condition has been assumed on the lumen side of the membrane, and hence, the resulting parabolic velocity, *ν_z_* (ms^−^^1^), has been assumed as follows:

(5) $$\nu_{z}\left(r\right)=2\bar{V}\left[1-{\left(\frac{r}{r_{1}}\right)}^{2}\right]$$

where *r*_1_ (m) is the inner radius of the lumen, and $\bar{V}\;$ is the average fluid velocity (ms^−^^1^) in the lumen of the fiber. The $\bar{V}\;$ is given below in Equation (6):

(6) $$\bar{V}=\frac{Q}{N\pi r_{1}^{2}}\ldots$$

Equation (6) shows that the average flow velocity is dependent on the feed flow rate, *Q* (m^3^ s^−^^1^), and the number of fibers, *N*, in the membrane contactor.

The initial condition when t = 0 and for all values of r and z where 0 < *r* < *r*_1_ and 0 < *z* < *L* is given in the following equation:

*C*_*l*_ = *C*_*o, in*_ (7)

where *C_o_* is the initial concentration (moles m^−3^) of H_2_S in the feed solution.

The boundary conditions are defined below for the transport of gases in a hollow fiber membrane contactor.

At *r* = 0, for all values of *t* (s) and *z* (m), the axial symmetry condition is applied as given below:

(8) $${\left(\frac{\partial C_{l}}{\partial r}\right)}_{r=0}=0$$

At *z* = 0, for all values of *r* and *t*,

(9) $$C_{l}=C_{tank}\;\left(t\right)$$

For *z* = *L*, for all values of r and t, the axial diffusion is assumed to be negligible compared to bulk convection flow, which is given by:

(10) $$D_{l}\left(\frac{\partial^{2}C_{l}}{\partial z^{2}}\right)=0$$

For the condition when *r* = *r*_1_, for all values of *z* and *t*, the flux of H_2_S in aqueous phase is equal to the diffusion of gases through the pores of the hollow fiber hydrophobic membrane as given below:

(11) $$D_{l}{\left.\frac{\partial C_{l}}{\partial r}\right|}_{r=r_{1}}=k_{g,\;pore}\;\left(\frac{p_{int}^{g}-p_{b}^{g}}{RT}\right)$$

where *p^g^_int_* and *p^g^_b_* are the partial pressures (Pa) of H_2_S gas at the lumen and shell side, respectively, while *k_g_*, R is the universal gas constant (J/(mol.K). Pore is the mass transfer co-efficient inside the pore of the hollow fiber membrane, and hence, the above equation becomes the following:

(12) $$D_{l}{\left.\frac{\partial C_{l}}{\partial r}\right|}_{r=r_{1}}=k_{g,\;pore}\;\left(\frac{p_{int}^{g}}{RT}\right)$$

At *r* = *r*_1_, Henry’s law can be applied at the liquid–gas interface.

*P*^*g*^_*int*_ = H_a_*C*_*l, int*_ (13)

where H_a_ is the Henry’s law constant (Pa.m^3^/mol).

The mass transfer coefficient inside the pore can be estimated by the following equation:

(14) $$k_{g,\;pore}=D_{c,\;pore}\left(\frac{\varepsilon }{\tau b}\right)$$

Equation (14) shows that mass transfer coefficient is dependent on the parameters, which include tortuosity, *τ*, of pore, porosity, *ε*, thickness, *b* (µm), of the membrane, and combined diffusivity, *D_c_*, pore.

It has been assumed that the transfer of the gas through the hydrophobic pores of the membrane occurs through both Knudsen and bulk diffusion. During Knudsen diffusion, pore size is smaller than the mean free path of the gas molecules and gas molecules colloid more with the walls of the pores than with each other. Due to excessive collisions with each other, the transport of the gas is impeded. The Knudsen diffusion coefficient, *D*_k_, pore, can be predicted from the kinetic theory by relating the pore diameter, d_pore_, with a mean free path of the gas.

(15) $$D_{k,\;\mathrm{pore}}=4850d_{pore}\sqrt{\frac{T}{M_{w}}}$$

where *M_w_* is the molecular weight of the gas. The unit of *D*_k_, pore is cm^2^ S^−^^1^. *T* is the temperature in *K* while d_pore_ is the pore diameter in nm.

In the case of bulk diffusion, the pore size is greater than the mean free path of the gas, which allows the gas to rapidly transfer based on the concentration gradient.

Hence, the removal of dissolved H_2_S in a membrane contactor through the pores of the membranes is based on both Knudsen and bulk diffusion as given below:

(16) $$\frac{1}{D_{c,\;pore}}=\frac{1}{D_{k,\;pore}}+\frac{1}{D_{air}}$$

where combined, Knudsen, and air diffusion coefficients are given in m^2^ s^−^^1^.

**Figure 11:**
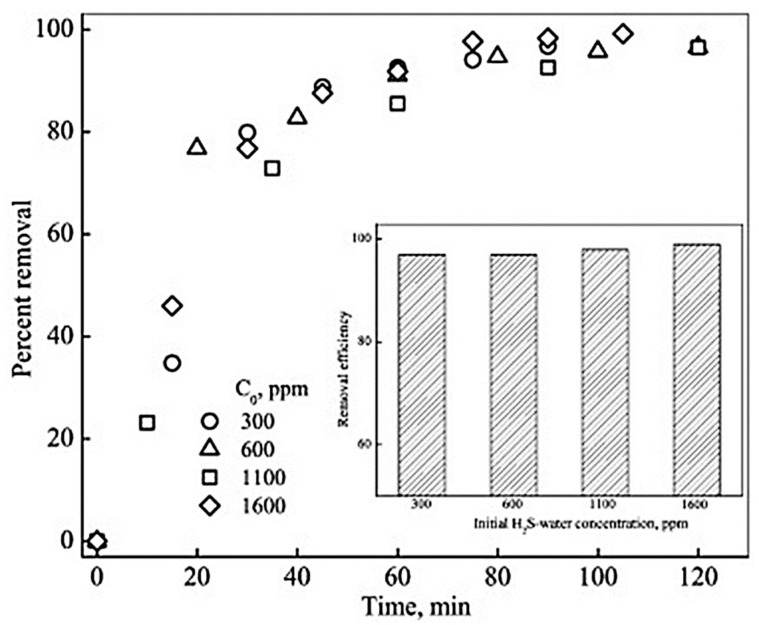
Variation in percent removal of H_2_Swith volume ofH_2_S-water passed for different initial H_2_S-water concentrations (Q = 0.2 L.min^−1^). Inset: Removal efficiency for different initial H_2_S–water concentrations at the end of operation. Adapted with permission from Ref. [31] (2013, Elsevier).

The above-mentioned models have been verified through simulations, and the predictions made by these models have been proven to be in complete agreement with the experimental results and observations. The following Figure 11 shows a higher removal of H_2_S at higher initial concentrations of H_2_S. The percent removal of H_2_S was found to be higher at the beginning of the operation with high H_2_S concentrations compared to lower H_2_S concentrations. However, with the passage of time, the removal efficiency reaches a steady state, and the removal of H_2_S flattens [31].

## 4. Challenges and Future Perspectives

Although several methods have evolved over the past few years for removing H_2_S from gaseous streams, very little attention has been paid to recovering dissolved H_2_S from wastewater resources. The currently used strategies also need extensive investigation and optimization for the efficient removal of dissolved H_2_S from wastewater streams. For example, the byproducts generated from the degradation of MEA-triazine are themselves a huge challenge, such as the amorphous polymeric byproduct apDTZ. The apDTZ deposits in the form of scales, which leads to the extensive fouling of the pipes and oil-producing facilities. Further investigations are required to ensure the complete utilization of the triazine scavengers. In addition to triazine-based scavengers, there is a need to search for novel types of scavengers that can eventually generate easily degradable byproducts.

The currently used methods for solubilizing scavenger byproducts are based on harsh oxidants, such as peroxy acids and hydrogen peroxides, which can cause excessive corrosion and damage to pipelines in the wastewater treatment plants. Although peroxy acids have been found useful for such applications, but the reaction is highly exothermic and spontaneous, which needs further investigation and optimizations. Control over the reaction is highly desirable for field applications. Moreover, the use of cosolvents for solubilizing the stubborn scavenger byproducts needs to be further explored to develop an efficient solvent system. The need of the day is to develop a strategy to minimize the generation of apDTZ.

Furthermore, the use of membranes for removing unspent scavengers and byproducts is also a potentially viable technique for industrial and large-scale applications. There is a need to develop efficient membranes with suitable surface chemistry to minimize membrane surface fouling. In addition, there is a need to explore different types of membranes, such as ceramic, polymeric, and composite membranes for removing scavenger byproducts from the water streams. In addition, different membrane configurations, such as hollow fiber and flat sheets, should be explored for removing scavenger byproducts from wastewater streams. Different membrane modules should also be designed and applied to remove scavenger byproducts with lower chances of membrane fouling.

The membrane contactors used for dissolved H_2_S removal are mainly focused on the use of a hydrophobic PVDF membrane and mostly employed in hollow fiber module form. There is a need to develop more hydrophobic membranes to minimize the loss of vapors through the pores of the membrane. The pores’ structure and size both need to be optimized to allow only the diffusion of H_2_S through the membrane. There is a need to develop an online system where the permeate of an RO plant should be directed towards a membrane contactor where the dissolved H_2_S can be efficiently removed from the permeate.

Since the percentage of dissolved H_2_S depends on the pH of the feed, there is a need to develop and optimize the conditions of the feed leading to less scaling and enhanced recovery of dissolved gas under operating conditions.

Hence, a proper investigation on the available techniques, including a monoethanol amine triazine (MEA-Trizine) scavenger, peroxyacetic acid oxidation, and membrane contactors, can resolve the serious challenge of scavenging the toxic H_2_S gas. The established Knudsen–Bulk diffusion model needs to be further optimized for a variety of hydrophobic membranes, leading to best performing membranes.

## 5. Conclusions

The current review has described the utilization of several techniques, including membrane-based separations for removing and recovering dissolved H_2_S from various feeds and wastewater streams. Among the various techniques that have been used for removing dissolved H_2_S, membrane contactors have shown huge potential for removing dissolved H_2_S. The hydrophobic membranes, such as PVDF and PTFE, have shown enormous potential in this regard because these membranes provide passageways to H_2_S gas while they do not allow water to permeate through them. Various mathematical models have been explored and developed for better understanding of gaseous diffusion through membrane contactors. Like membrane-based separations, triazine scavengers have also shown huge capabilities for treating dissolved H_2_S from wastewater resources and, hence, have been used for sweetening of sour water. However, the H_2_S scavengers generate several byproducts that polymerize and become a cause of concern. Hence, the use of some oxidizing agents, such as per(oxy) acids, has been shown to be promising for treating the byproducts generated during the scavenging action of H_2_S scavengers. Hence, the exploration of viable and commercially scalable dissolved H_2_S treating technologies is desperately required to mitigate excessive corrosion and lower the risk of acute toxicity caused by dissolved H_2_S.

## Data Availability

Data sharing not applicable.

## Supplementary Materials

The following supporting information can be downloaded at: https://www.mdpi.com/article/10.3390/membranes13070646/s1, Figure S1: (Left) Example of amorphous polymeric dithiazine deposition in field installa-tions, note the very pervasive and solid form and (right) crystallization of monomeric dithiazine which will remain in this form for only a few hours before spontaneously polymerizing to form apDTZ [25]; Figure S2: (a) First double SN2 reaction and substitution of the first nitrogen atom with sulfur to yield the formation of thiadiazine, (b) Second double SN2 reaction and substitu-tion of the second nitrogen atom with sulfur to yield the formation of dithiazine, (c) (Left) Bisulfide anion nucleophilic attack on thiadiazine, resulting in the C–N bond breaking, and (right) bisulfide anion nucleophilic attack on dithiazine which is ineffective and, in this instance, cannot break the C–N bond and (d) MEP surfaces for (left) thiadiazine and (right) dithiazine with nucleophilic center and carbon positive density indicated [28].

## Abbreviations

H_2_S	Hydrogen sulfide
PVDF	Polyvinylidene difluoride
MEA-Triazine	Monoethanol amine triazine
PW	Produced water
WHO	World Health Organization
RO	Reverse Osmosis
Ppm	Parts per million
CO_2_	Carbon dioxide
HET	1,3,5-tri(2-hydroxyethyl)hexahydro-S-traizine
DTZ	5-(2-hydroxyethyl)hexahydro-1,3,5-dithiazine
MEP	Molecular electrostatic potential
apDTZ	Amorphous polymeric dithiazine
NF	Nanofiltration
TOC	Total organic content
SUS	Spent and unspent scavenger
TFC	Thin film composite
IP	Interfacial polymerization
MPD	meta-phenylenediamine
TMC	Trimesoyl chloride
PSf	Polysulfone
PET	Polyethylene terephthalate
HFMCs	Hollow fiber membrane contactors
PP	Polypropylene
MD	Membrane distillation
M-DCMD	Modified direct contact membrane distillation
2S-AnMBR	Two-stage anaerobic membrane bioreactor
SAF-MBR	Staged anaerobic fluidized membrane bioreactor
COD	Chemical oxygen demand
