# Supplementary material for: Recovery of Dissolved Hydrogen Sulfide from Various Wastewater Streams Using Membranes and Other Relevant Techniques: A Review
Source: Membranes (Basel). 2023 Jul 5;13(7):646. doi: 10.3390/membranes13070646 (PMC10385389; doi:10.3390/membranes13070646)
Supplement: Supplementary file 1 [file membranes-13-00646-s001.zip › membranes-2458022-supplementary.pdf]

Supplementary file

Recovery of Dissolved Hydrogen Sulfide from Various Wastewater Streams using Membranes and Other Relevant Techniques: A Review

Abdul Waheed [1,*], Isam H. Aljundi [1,2] and Umair Baig [1,*]

[1] Interdisciplinary Research Center for Membranes and Water Security, King Fahd University of Petroleum and Minerals, Dhahran 31261, Saudi Arabia
[2] Chemical Engineering Department, King Fahd University of Petroleum and Minerals, Dhahran 31261, Saudi Arabia
* Correspondence: abdul.waheed@kfupm.edu.sa (A.W.); umairbaig@kfupm.edu.sa (U.B.)

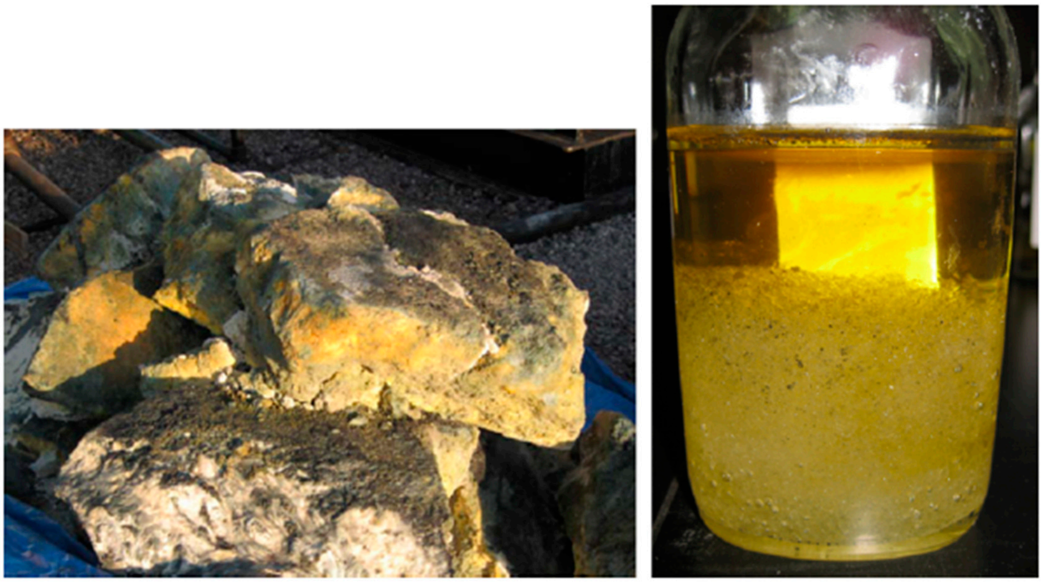

Figure S1: (Left) Example of amorphous polymeric dithiazine deposition in field installa-tions, note the very pervasive and solid form and (right) crystallization of monomeric dithiazine which will remain in this form for only a few hours before spontaneously polymerizing to form apDTZ [1].

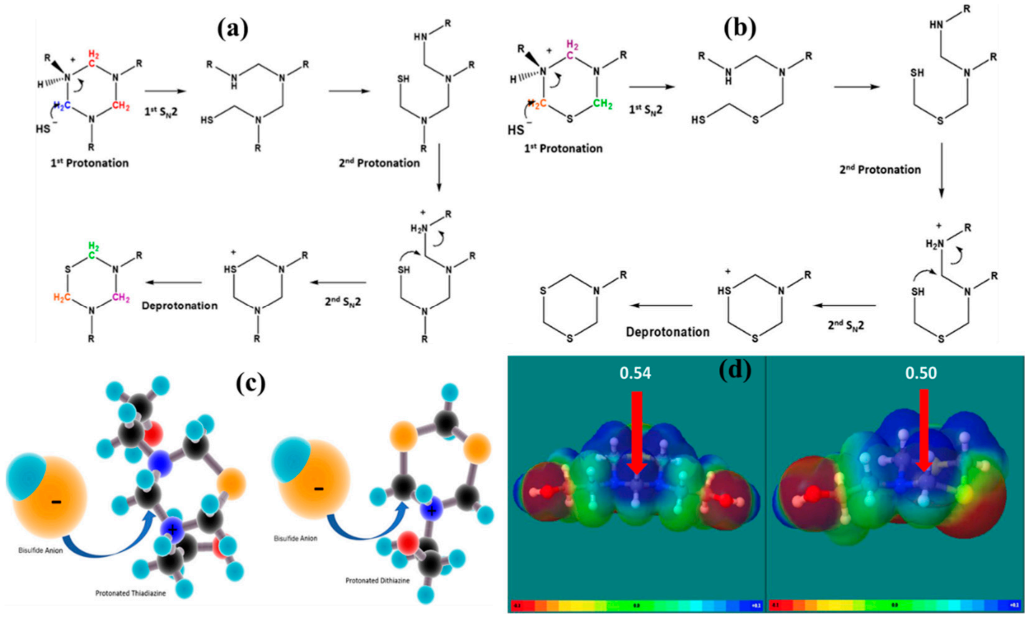

Figure S2: (a) First double SN2 reaction and substitution of the first nitrogen atom with sulfur to yield the formation of thiadiazine, (b) Second double SN2 reaction and substitu-tion of the second nitrogen atom with sulfur to yield the formation of dithiazine, (c) (Left) Bisulfide anion nucleophilic attack on thiadiazine, resulting in the C–N bond breaking, and (right) bisulfide anion nucleophilic attack on dithiazine which is ineffective and, in this instance, cannot break the C–N bond and (d) MEP surfaces for (left) thiadiazine and (right) dithiazine with nucleophilic center and carbon positive density indicated [2].

References

1. Taylor, G.N. An Example of Chemical Recycling in the Oil and Gas Industry - A By-Product from Hydrogen Sulphide Scavenging Is Identified, Isolated and Formulated into a Highly Effective Corrosion Inhibitor. Proc. - SPE Int. Symp. Oilf. Chem. 2011, 1, 73–84, doi:10.2118/140439-MS.
2. Wylde, J.J.; Taylor, G.N.; Sorbie, K.S.; Samaniego, W.N. Formation, Chemical Characterization, and Oxidative Dissolution of Amorphous Polymeric Dithiazine (ApDTZ) during the Use of the H2S Scavenger Monoethanolamine-Triazine. Energy & Fuels 2020, 34, 9923–9931, doi:10.1021/ACS.ENERGYFUELS.0C01402.
